# The Potential of Collagen Treatment for Comorbid Diseases

**DOI:** 10.3390/polym15193999

**Published:** 2023-10-05

**Authors:** Hsiuying Wang

**Affiliations:** Institute of Statistics, National Yang Ming Chiao Tung University, Hsinchu 300093, Taiwan; wang@stat.nycu.edu.tw

**Keywords:** collagen, disease, inflammation, tissue damage, treatment

## Abstract

Collagen, the most abundant protein in our bodies, plays a crucial role in maintaining the structural integrity of various tissues and organs. Beyond its involvement in skin elasticity and joint health, emerging research suggests that collagen may significantly impact the treatment of complex diseases, particularly those associated with tissue damage and inflammation. The versatile functions of collagen, including skin regeneration, improving joint health, and increasing bone strength, make it potentially useful in treating different diseases. To the best of my knowledge, the strategy of using collagen to treat comorbid diseases has not been widely studied. This paper aims to explore the potential of collagen in treating comorbid diseases, including rheumatoid arthritis, osteoarthritis, osteoporosis, psoriatic arthritis, sarcopenia, gastroesophageal reflux, periodontitis, skin aging, and diabetes mellitus. Collagen-based therapies have shown promise in managing comorbidities due to their versatile properties. The multifaceted nature of collagen positions it as a promising candidate for treating complex diseases and addressing comorbid conditions. Its roles in wound healing, musculoskeletal disorders, cardiovascular health, and gastrointestinal conditions highlight the diverse therapeutic applications of collagen in the context of comorbidity management.

## 1. Introduction

Collagen, a vital protein synthesized within the body, is a fibrillar protein and is the primary structural component in the skin, tendons, and bone. Collagen constitutes a major component of the conjunctive and connective tissues, providing mechanical stability, elasticity, and strength to organisms [1,2]. The extracellular matrix (ECM) is the non-cellular component of all tissues, shaping the physical environment surrounding cells, and plays crucial roles in both providing structural support and facilitating cellular signaling processes [3]. In the human body, collagen constitutes one-third of the total protein content, accounts for approximately three-quarters of the dry weight of the skin, and stands as the predominant constituent within the ECM [4].

Collagen has a unique structure formed with three polypeptide chains (α-chains) that wind around each other to create a strong triple helix. Each of these chains has a specific repeating sequence (Gly-X-Y), where Gly is glycine, and X and Y are often proline and hydroxyproline, which makes it similar to a polyproline helix. This triple helical arrangement gives collagen its distinctive properties, contributing to its strength and function in various tissues [5]. The distinct collagen structure may play a significant role in ensuring its mechanical stability.

Collagen exhibits a variety of applications and demonstrates positive medical effects [6,7]. The superior properties of collagen-based biomaterials include biocompatibility, biodegradability, mechanical strength, and cellular activity. These attributes render collagen highly suitable for various biomedical applications, including wound healing, tissue engineering, surface coating of medical devices, and skin supplementation [8]. Collagen-based supplements have emerged as a fundamental component in addressing the effects of the aging process, demonstrating their established capacity to repair skin damage and bestow a rejuvenated and healthy appearance sought after in the pursuit of beauty [9]. Collagen has been recognized as a drug carrier possessing numerous benefits, including non-toxicity, superior biocompatibility, and favorable interactions with pharmaceutical agents [10,11]

Collagen could impact tumor cell behavior by interacting with integrins, discoidin domain receptors, tyrosine kinase receptors, and various signaling pathways, and extensive collagen deposition is the primary pathological hallmark of certain cancers [12,13].

Comorbid diseases refer to the simultaneous presence of two or more medical conditions in an individual. Collagens are implicated in the pathogenesis of a multitude of diseases, and the observed associations among these disorders indicate a compelling association between the comorbidity of these conditions and an underlying deficiency in collagen [6].

In this paper, the potential of collagen treatment for comorbid diseases is discussed.

## 2. Types and Sources of Collagen

There are five primary types of collagen, which are classified as types I–V. Table 1 presents the tissues or organs composed of the five predominant types of collagen.

Type I collagen is the most abundant form of collagen in the human body [14] and acts as the primary organic scaffold, undergoing mineralization to support the process of bone development. This collagen is continuously synthesized and deposited in the ECM and subsequently broken down by enzymes in a finely balanced cycle that enables growth. During development, the turnover of collagen is rapid, which later stabilizes during adulthood. However, in later life, collagen turnover increases once more to counteract the cumulative detrimental effects associated with chronological aging and photoaging [9]. The principal components comprising the EMC of cartilage consist of type II collagen, proteoglycans, and several proteins, which include certain minor collagens [15]. Loss of type II collagen led to an acceleration of chondrocyte hypertrophy, mediated with the BMP-SMAD1 signaling pathway [16].

Type III collagen is extensively cross-linked with type II collagen, playing a vital role in regulating the fibrillar structure and biomechanical properties of cartilage tissue [17]. It is expressed during early embryonic development and remains prevalent throughout the process of embryogenesis. Type III collagen plays a crucial role in facilitating the proper fibrillogenesis of type I collagen in various organs. Type I and III collagens are two major subtypes in the female pelvic tissues, with type I collagen affecting tissue stiffness, while type III collagen is related to tissue elasticity [18].

Type IV collagen, being the principal collagen constituent of the basement membrane, plays a vital role in forming a network that underlies both epithelial and endothelial cells, functioning as a critical partition between tissue compartments. This collagen type is extensively involved with various binding partners, forming the foundational structure of the basement membrane [19].

Type V collagen plays a role in modulating the formation of fibrils and tissue characteristics, thereby contributing to the composition of the bone matrix, corneal stroma, and interstitial matrix of muscles [20]. Type V collagen is typically found in association with type I collagen. It helps control the diameter and organization of collagen fibrils, thus influencing the overall strength and stability of tissues.

The most common sources of collagen used in biomaterial or biomedicine applications include human collagen, as well as collagen derived from bovine, porcine, and marine organisms. Collagen could be readily obtained from animal tissues, particularly bones [21]. Fibrillar collagen extracted from a bovine Achilles tendon demonstrated the potential for creating 3D printed scaffolds [22]. Collagen extracted from an ovine Achilles tendon exhibited a significant enhancement in human dermal fibroblast attachment and proliferation [23]. Marine organisms and their wastes could be a sustainable, environmentally friendly collagen source for various applications [24]. The mechanical characteristics of marine-derived collagen exhibited a general inferiority when compared to those of porcine-derived collagen in tissue engineering [25].

Although animals make up the majority of collagen sources used in biomaterial science, the occurrence of outbreaks of bovine spongiform encephalopathy, transmissible spongiform encephalopathy, and foot-and-mouth disease in recent decades has resulted in restrictions and limitations on the utilization of collagens from these particular sources [26]. Furthermore, animal-derived collagen has several drawbacks, such as immunogenicity, batch-to-batch variation, and the risk of pathogenic contamination. In light of these challenges, recombinant collagen might be a promising resolution to address these concerns [27].

## 3. Collagen Treatment

Collagen treatment offers a range of advantages, including antioxidant and anti-inflammatory properties, wound healing support, benefits for hair and nail health, improvement in gut health, support for joint health, skin rejuvenation, and potential support for musculoskeletal disorders and cardiovascular health.

The impact of collagen peptides isolated from milkfish scales, known as milkfish scale collagen peptides (MSCP), and extracted using the pepsin-soluble collagen method on cell viability was investigated [28]. MSCP exhibited anti-inflammatory effects by reducing lipoxygenase activity and nitric oxide radicals. MSCP demonstrated strong antioxidant properties as indicated with its ability to scavenge DPPH· and ABTS·+ radicals, as well as reduce cellular reactive oxygen species. An approach known as Subcision was introduced as a minimally invasive technique to treat scars, involving the use of extremely fine needles to disrupt dermal collagen and stimulate dermal remodeling and skin resurfacing [29,30]. The efficacy of the microneedling-delivered irradiated amniotic collagen matrix (IACM) compared to platelet-rich plasma (PRP) in facial rejuvenation was investigated [31]. The result showed that both approaches demonstrated effectiveness and safety in treating skin aging. However, microneedling with IACM yielded superior results compared to PRP, clinically, pathologically, and through an Antera camera analysis.

Collagen can decrease the levels of matrix metalloproteinases (MMPs) by acting as a sacrificial substrate for excessive proteases in chronic wounds. Targeting broad-spectrum excessive MMP levels through collagen dressings could potentially have a positive effect on the healing rates of challenging wounds [32]. The primary health benefits of collagen are associated with dermatological and orthopedic conditions [33]. The effects of bioactive collagen peptide intake on hair thickness and metabolism were investigated [34]. After the consumption of bioactive collagen peptides, a notable increase in hair thickness was observed, which could be attributed to a direct impact of the supplemented peptides on hair metabolism.

The efficacy and tolerability of undenatured type II collagen (UC-II) in regulating joint function and joint pain resulting from strenuous exercise in healthy subjects were investigated with a randomized, double-blind, and placebo-controlled study on healthy volunteers [35]. The findings indicated that the daily intake of 40 mg of UC-II was well tolerated and resulted in enhanced knee joint extension among healthy participants.

Purified porcine atelocollagen (PAC) showed promise as a potential treatment option for managing refractory chronic musculoskeletal pain in a study that recruited patients with chronic refractory pain, where musculoskeletal damage or defects were suspected based on evidence from imaging studies [36]. PAC has the potential to facilitate tissue recovery, act as a scaffold for repair, or directly alleviate inflammation. A new recombinant human type III collagen (hCOLIII) with thromboprotective properties was developed for cardiovascular stents [37]. It supported endothelial cell growth, inhibited smooth muscle cell proliferation, and enhanced healing. Injectable recombinant human collagen type I (rHCI) and type III (rHCIII) matrices were used to treat myocardial infarction in a mice study, restoring mechanical properties, reducing scar size, and preventing heart enlargement [38]. rHCI could promote healing, enhance cardiomyocyte survival, and reduce pathological remodeling.

Beyond treating individual diseases, collagen can be used to treat different diseases simultaneously, including comorbid diseases. To explore the benefits of collagen treatment in comorbid diseases, I initially conducted a search to identify diseases related to collagen treatment in various databases, including PubMed and Google Scholar. This systematic search utilized the following terms: ‘collagen, diseases’, ‘collagen treatment’, and ‘collagen therapy’.

Subsequently, for every pair of disorders among the identified diseases, I utilized PubMed and Google Scholar to search the literature to investigate whether there was a relationship between the two disorders. If an association was found between the two diseases, they were considered comorbid diseases for this research.

**Figure 1:**
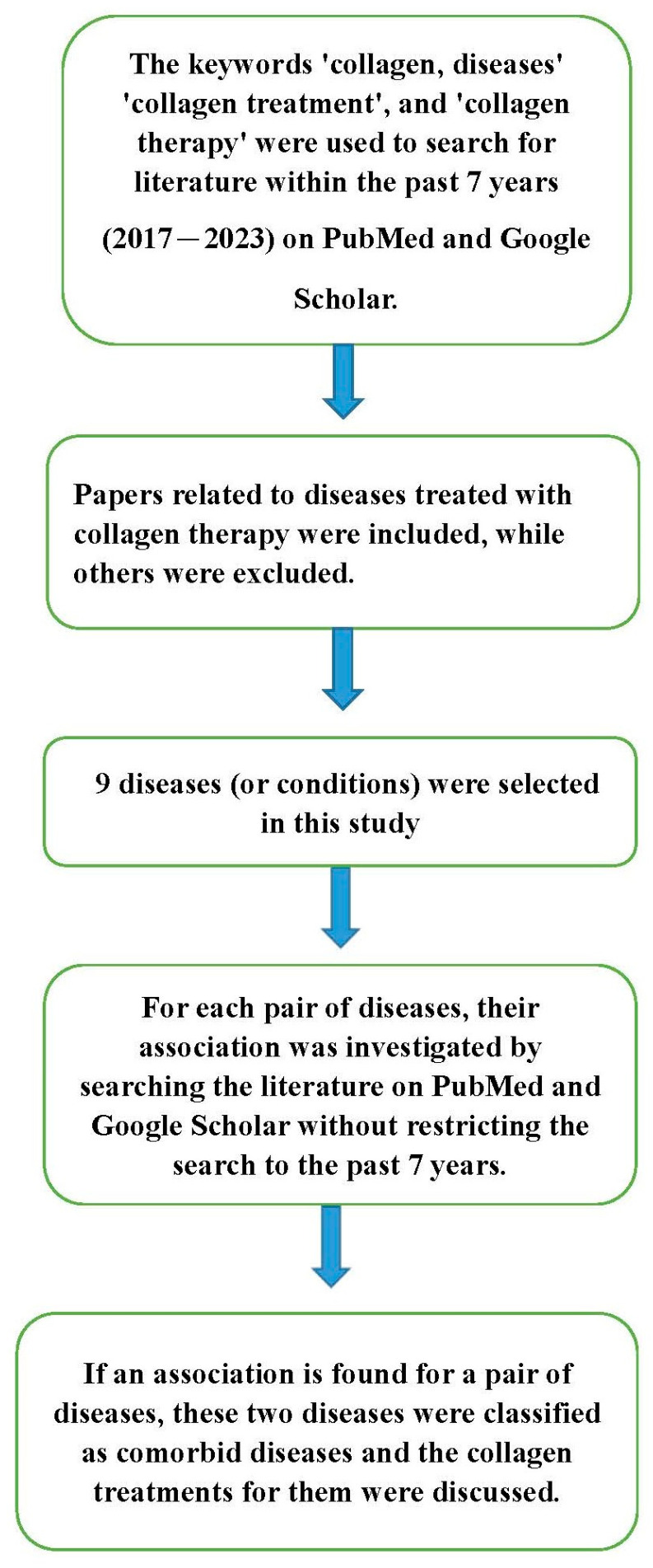
The flow chart of the methodology.

The methodology flowchart is provided in Figure 1. The details of collagen treatment for comorbid diseases are provided in the next section.

## 4. Comorbid Diseases’ Treatment

Comorbidity can be observed between rheumatoid arthritis (RA) and osteoarthritis (OA), as both are chronic joint disorders that can coexist in a patient [39]. In addition, dermatological patients may receive local or systemic treatments involving glucocorticoids and immunosuppressants, which could elevate the likelihood of osteoporosis [40].

### 4.1. Rheumatoid Arthritis, Bone Regeneration, and Osteoporosis

There are many types of arthritis. Some of the most common types include RA, OA, psoriatic arthritis, gout, juvenile idiopathic arthritis, ankylosing spondylitis, and systemic lupus erythematosus. RA is a systemic autoimmune disorder primarily impacting the joints, and it tends to have a higher prevalence in industrialized nations [41]. The posttranslational modifications of type II collagen might contribute to RA chronicity by creating new epitopes during the process of inflammation [42]. 

The causative factors of degenerative bone conditions are linked to various diseases, such as RA and osteoporosis [43]. Osteoporosis is a significant public health concern, characterized by the deterioration of bone tissue’s microarchitecture and decreased bone mineral density [44]. These factors contribute to diminished bone strength, heightened bone fragility, and an elevated susceptibility to skeletal fractures. The prevalence of osteoporosis among individuals diagnosed with RA is roughly twice as high compared to the general population within the same age range [45]. Osteoporosis poses a significant health concern among women in the postmenopausal stage of life, and the mechanism of osteoporosis is the age-related decline in hormonal/estrogen levels [46].

Oral tolerance is a favorable therapeutic approach for RA due to its minimal side effects and straightforward clinical application. Oral administration of type II collagen suppressed IL-17-associated RANKL expression of CD4+ T cells in collagen-induced RA [47].

Marine collagen and its derivatives have demonstrated efficacy and utility in the prevention and treatment of osteoporosis. Collagen can also potentially address other bone-related diseases by promoting bone mineral density, mineral deposition, and crucially, the maturation and proliferation of osteoblasts [48]. A study investigated the anabolic effects of specific collagen peptides (SCP) on bone formation and bone mineral density (BMD) [49]. The consumption of SCP was found to enhance BMD in postmenopausal women experiencing age-related decline in BMD. Collagen-enriched diets were investigated for their potential benefits on bone health. An innovative approach involving hydrolyzed collagen ingestion and a blood sample analysis revealed direct effects on human and mouse bone cells, reducing bone loss in a post-menopausal osteoporosis mouse model [50]. These findings suggest that hydrolyzed collagen offers osteoporosis prevention benefits beyond protein supplementation.

### 4.2. Osteoarthritis, Bone Regeneration, and Osteoporosis

Apart from RA and osteoporosis, OA stands as one of the prevalent chronic orthopedic diseases [51]. OA is a degenerative condition characterized by the progressive deterioration of joint cartilage and remodeling of the surrounding bone. 

The administration of oral collagen decreases the symptoms of OA [52]. A hydrolyzed (<3 kDa) bovine collagen injectable formulation, CHondroGrid, has been demonstrated to be a safe and effective adjuvant in the treatment of symptomatic knee OA [53].

There are two forms of orally administered collagen for OA treatment: undenatured type II collagen (UC-II) and partially hydrolyzed collagen. UC-II maintains the collagen’s natural biological activity, while the latter involves enzymatic, heat, or pH degradation of collagen [54]. Thirty patients with knee OA and swollen joints were included in a study to evaluate the effect of an oral preparation containing a naturally occurring matrix of hydrolyzed collagen type II, chondroitin sulfate, and hyaluronic acid, and bioactive oligopeptides of natural hydrolyzed keratin [55]. The treated group showed a reduction in IL-8, IL-6, and IL-10 levels. Despite the possible beneficial effects of collagen supplementation, studies have also shown adverse effects of collagen supplementation in patients with RA or OA, as well as a low efficiency of collagen treatment compared to routine treatments [56]. 

### 4.3. Psoriatic Arthritis, Bone Defects, and Osteoporosis

Psoriatic arthritis (PsA) is an inflammatory joint disease that typically affects individuals with psoriasis, causing joint pain, stiffness, and swelling. Levels of biomarkers associated with type I collagen degradation and type III collagen degradation are elevated in patients with PsA compared to healthy individuals [57]. The comorbidities of PsA include cardiovascular disease, metabolic syndrome, obesity, diabetes mellitus, dyslipidemia, inflammatory bowel disease, fatty liver disease, uveitis, kidney disease, infections, osteoporosis, depression, central sensitization syndrome, and gout [58]. Gout is a systemic disorder characterized by the accumulation of monosodium urate crystals in various tissues [59]. Chronic tophaceous ulcers in patients with tophaceous gout are infrequent but pose treatment challenges [60]. Various therapeutic approaches are employed to address tophaceous ulcers, such as the application of a petroleum-jelly-based solution containing 3% citric acid topically, and heterologous lyophilized collagen [60]. A drug delivery system was developed using pH-sensitive hydrogel beads composed of fish scale collagen and carrageenan, which was to enhance the bioavailability of allopurinol, a medication used in the treatment of gout [61]. 

### 4.4. Sarcopenia and Gastroesophageal Reflux

Sarcopenia is a progressive skeletal muscle disorder, leading to loss of muscle mass and muscle strength over time, that is associated with various negative consequences such as falls, functional decline, frailty, and mortality [62]. Sarcopenia was identified as a predictive factor for gastroesophageal reflux disease (GERD), and sarcopenic obesity was found to be a predictive factor for erosive reflux disease. GERD is a disorder that arises when the backflow of stomach contents leads to distressing symptoms or complications in the esophagus or beyond [63]. A study investigated the association between sarcopenia and GERD, involving a total of 3414 patients diagnosed with GERD, of whom 574 (16.8%) had sarcopenia [64]. A prospective 5-year longitudinal study on 178 individuals was conducted to find the risk factors of GERD, and the result showed that sarcopenia was a significant risk factor for the development of GERD [65]. 

Sarcopenia can decrease muscle strength. The association between prevalent GERD and muscular strength in older adults was studied, and the outcome revealed that muscular strength was independently and inversely associated with GERD in older adults [66]. Back muscle strength is an important risk factor for the development of GERD symptoms [67]. Sarcopenia might decrease back muscle strength and cause GERD. Sarcopenia and degeneration of back muscles are considered risk factors for degenerative adult spinal deformity, leading to sagittal imbalance and degenerative spinal diseases [68]. 

Both the collagen tripeptide (CTP) and collagen peptide (CP) supplements were observed to enhance the expression of different proteins, with CTP increasing IGF-1, PI3K/AKT, and mTOR, and CP increasing IGF-1 and AMPK, in the gastrocnemius of aging mice [69]. These distinct mechanisms of action for CP and CTP supplements contribute to the amelioration of age-associated sarcopenia. CP supplementation following resistance exercise training has been demonstrated to enhance muscle mass and strength [70]. Following 12 weeks of hypertrophy resistance exercise training with collagen supplementation compared to a placebo, significantly higher body mass (BM) and fat-free mass (FFM) were observed, along with a slightly more pronounced increase in strength in the collagen group compared to the placebo group.

GERD might be induced with decreased esophageal sphincter pressure. The usage of collagen in pharmaceutical and surgical treatments for GERD has been developed [6]. Patients experiencing severe and treatment-resistant reflux symptoms underwent endoscopic treatment [71]. A cross-linked bovine dermal collagen injection was administered beneath the mucosa in the lower esophageal sphincter region, resulting in objective evidence of reduced reflux in the treated patients. Polymethylmethacrylate microspheres suspended in bovine collagen demonstrated remarkable potential as an injectable implant material for augmenting the lower esophageal sphincter in GERD endoscopic therapy [72].

During an 8-week investigation, the ingestion of collagen peptide supplements potentially led to decreased GERD and bloating, along with enhanced bowel frequency, without the incorporation of any additional dietary or lifestyle interventions or guidance [73]. The effect of a type of functional food, which included marine collagen peptides and other ingredients, on GERD patients was investigated, and the result showed that the functional food could serve as adjuvant therapy in GERD patients [74]. This functional food meal could decrease the percentage of time with pH < 4 in the gastric body and increase the mean pH in both the gastric fundus and gastric body when compared to the high-fat and standard meals. 

### 4.5. Sarcopenia and Periodontitis

Periodontitis is a chronic, multifactorial inflammatory condition associated with the accumulation of dental plaque. It is characterized by the gradual degradation of the supporting structures of the teeth, including the periodontal ligament and alveolar bone [75]. Poor oral health could have an impact on food selection and nutrient intake, ultimately leading to malnutrition, frailty, and sarcopenia [76].

The uses of collagen in periodontal regeneration include hemostatic plugs/sponges to control bleeding, resorbable oral wound dressings for faster healing and graft/extraction site closure, and collagen membranes as protective barriers promoting regenerative potential in periodontal and implant procedures [77]. Fish collagen and polyvinyl alcohol (Col/PVA) dual-layer membranes were developed using a combined freezing/thawing and layer coating method for guided tissue regeneration in the treatment of periodontal tissue defects [78].

In periodontal recall patients, the additional consumption of targeted collagen peptides has the potential to further augment the anti-inflammatory impact of professional mechanical plaque removal [79]. A rat study suggested that the whey component of milk protein promoted bone collagen synthesis, improved bone strength, and prevented alveolar bone loss by elevating hydroxyproline levels, thereby enhancing bone integrity [80,81]. Consuming dairy foods frequently (≥7 servings/week) was linked to a 24% lower prevalence of periodontal disease when compared to those who never consumed them [82]. The impact of dairy proteins on sarcopenia-related functions in middle-aged and older adults was examined [83]. The use of dairy protein as a potential nutritional strategy could enhance appendicular muscle mass in middle-aged and older adults.

### 4.6. Skin Aging and Osteoporosis

The skin is a complex organ, comprising three areas: the epidermis layer, dermis layer, and subcutaneous tissue [84]. The dermis primarily consists of the ECM produced by fibroblasts, encompassing connective tissue containing glycosaminoglycans, proteoglycans, structural proteins like collagen and elastin, and specific macromolecules like fibrin and hyaluronic acid [85]. The skin undergoes a progressive decline in its morphological and physiological attributes as age increases, making it the initial conspicuous manifestation of aging [86].

The close relationship between skin collagen and bone density is evident [87]. Patients with Cushing’s syndrome have reduced skin collagen and bone density, while those with acromegaly have increased skin collagen and denser bones. Conversely, individuals with hypopituitarism show reduced skin collagen and thinner bones. The outcomes of a meta-analysis demonstrated favorable effects of hydrolyzed collagen supplementation over a placebo, particularly concerning improvements in skin hydration, elasticity, and reduction in wrinkles [88]. The superiority of marine collagen compared to collagen derived from land animals, along with its biomedical applications in bone and skin repair, has been demonstrated [48].

### 4.7. Diabetes mellitus and osteoporosis

Type 1 diabetes mellitus (DM) contributes to aggravated bone loss in osteoporotic patients [89]. Osteoporosis frequently occurs in individuals with type 2 DM. Advanced age and female gender have been shown to be associated with a higher prevalence of osteoporosis [90].

Reduced enzymatic cross-linking and/or elevated non-enzymatic cross-link formation within bone collagen have been suggested as contributing factors to compromised bone mechanical characteristics observed in aging, osteoporosis, and DM [91].

The effect of marine collagen peptides on glucose metabolism and insulin resistance, utilizing a rat model of type 2 DM, was investigated [92]. Certain doses of marine collagen peptides (equal to or exceeding 4.5 g/kg body weight/day) demonstrated enhanced glucose metabolism and improved insulin resistance.

A study conclusively demonstrated the effectiveness of collagen-derived peptides as an adjunctive supplement in the management of type 2 DM [93]. The study subjects who received oral supplementation of collagen-derived peptides showed a significant reduction in fasting blood glucose (FBG) and HbA1c levels, bringing them back to normal levels by the end of the study period. The protective role of marine collagen peptides on carotid artery vascular endothelial cells (CAVECs) in type 2 DM and the underlying mechanism were studied [94]. Diabetic Wistar rats were divided into groups receiving different marine collagen peptide doses, along with a control group. Marine collagen peptide treatment in rats for 4 weeks reduced blood glucose levels and alleviated endothelial thinning and inflammatory exudation in CAVECs. It suggested that a moderate oral marine collagen peptide dose (≥4.5 g/kg body weight/day) could serve as a novel therapeutic approach for protecting against early cardiovascular complications related to type 2 DM. 

**Figure 2:**
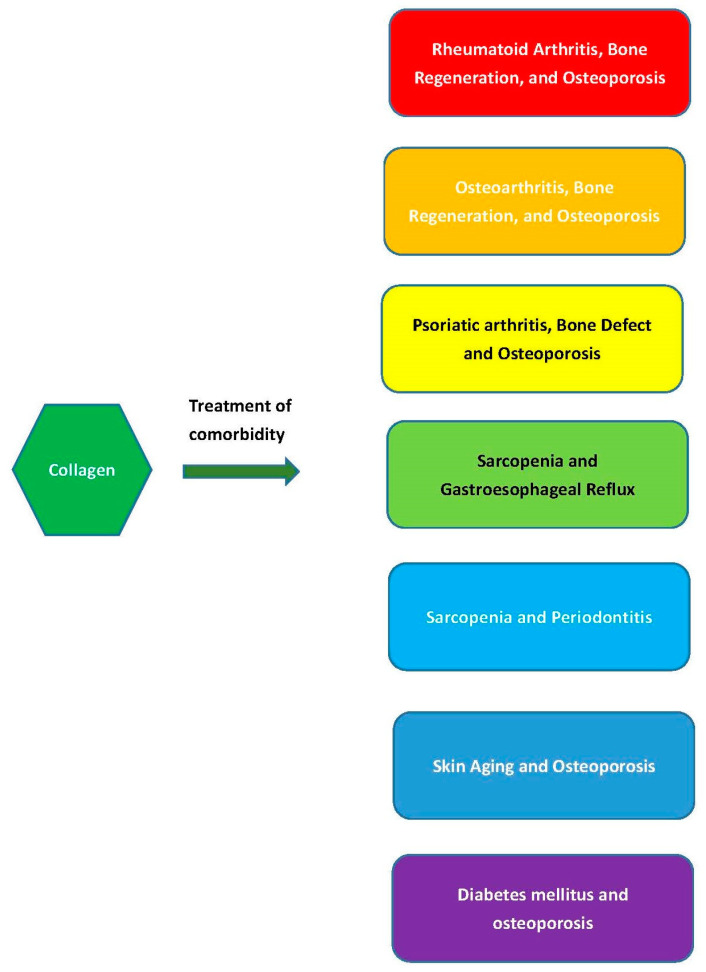
Collagen intake or treatment simultaneously benefiting comorbid conditions.

The comorbid conditions discussed in this section is summarized in Figure 2.

## 5. Discussion

Collagen is an essential component in our body, and its consumption through moderate daily supplements can be beneficial. Apart from the comorbid diseases discussed in Section 3, collagen deficiency may be the cause of other comorbid conditions.

**Figure 3:**
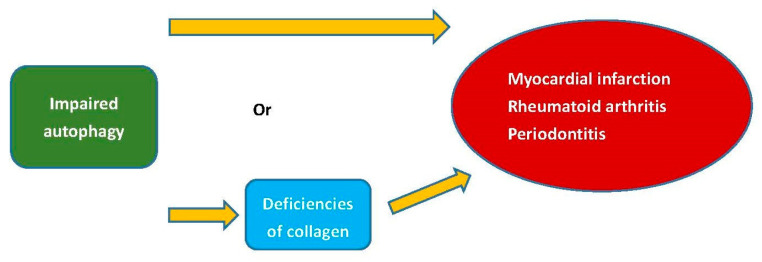
The deficiencies of collagen or autophagy could potentially contribute to the pathogenesis of myocardial infarction, RA, and periodontitis.

Individuals diagnosed with RA experience cardiovascular events at a rate that is 1.5 to 2 times higher than that of the general population. Additionally, cardiovascular events are a primary cause of mortality among RA patients [95]. Type II collagen, as an autoantigen, is sufficient to induce arthritis [96]. Myocardial infarction, a type of cardiovascular disease, is an ischemic condition causing cardiac tissue necrosis due to coronary artery occlusion. The size of scar tissue after myocardial infarction could independently predict cardiovascular outcomes, and the absence of type V collagen could modify the mechanical characteristics of the scar tissue [97]. Periodontitis was demonstrated to be linked to RA. There exists a significant association between P. gingivalis periodontitis, anti-P. gingivalis antibodies, and RA [98]. Autophagy governs the synthesis of type I collagen in human-tooth-supportive periodontal ligament cells by removing misfolded proteins [99]. Additionally, impaired autophagy has been found to play a role in the development of myocardial infarction and RA [99]. The deficiencies of collagen caused by impaired autophagy could potentially contribute to the pathogenesis of myocardial infarction, RA, and periodontitis (Figure 3).

The global outbreak of coronavirus disease 2019 (COVID-19) originated from severe acute respiratory syndrome coronavirus 2 (SARS-CoV-2), marking the emergence of a pandemic since late 2019 [100,101]. COVID-19 has resulted in a variety of neurological or other complications [102,103]. Collagen treatment is also available for COVID-19. A double-blind, randomized, placebo-controlled clinical trial was conducted to assess the safety and effectiveness of intramuscularly administered polymerized type I collagen (PTIC) in adult symptomatic COVID-19 outpatients [104]. The findings indicated that the administration of intramuscular PTIC during the initial treatment week was associated with a reduction in various severe COVID-19 biomarkers among symptomatic outpatients. COVID-19 is associated with hyperinflammation, and recently, the potential of porcine collagen has been explored for the treatment of COVID-19-induced hyperinflammation [105].

Many of the comorbid diseases discussed in Section 3 are also linked to COVID-19. As a result, collagen therapy could potentially offer concurrent benefits for both these diseases and COVID-19. Individuals with SARS-CoV-2 infection are more susceptible to experiencing gastrointestinal disturbances during the later stages of COVID-19 [106]. Consequently, the post-COVID-19 care approach should encompass a focus on maintaining and addressing gastrointestinal well-being and conditions. The collagen treatment for GERD in COVID-19 is suggested as an alternative to some other GERD drugs. The utilization of collagen therapy for GERD in COVID-19 was proposed as a viable substitute for certain other pharmaceutical options targeting GERD [6]. Individuals with a predisposition to RA are at an elevated risk of infection compared to the general population, partly due to the iatrogenic effects of drug therapies associated with RA. As a result, the COVID-19 pandemic could pose an increased health emergency risk for RA [107]. DM is linked to COVID-19. COVID-19 patients with DM or hyperglycemia exhibit mortality and severity of the disease two to four times higher than those without DM [108]. The potential for acute sarcopenia is expected to be most pronounced among elderly patients with COVID-19 [109]. A case–control study was conducted to investigate the correlation between periodontitis and complications arising from COVID-19 [110]. The findings indicated that periodontitis was linked to an elevated risk of COVID-19 patients requiring intensive care unit (ICU) admission and assisted ventilation, and experiencing mortality, along with elevated blood levels of biomarkers associated with more unfavorable disease outcomes. The alteration in the prevalence of osteoarthritis among elderly individuals in Bangladesh amid the COVID-19 pandemic was investigated [111]. This implies that the intake of nutrients or supplements for collagen synthesis could potentially contribute to mitigating comorbidities in COVID-19. While collagen offers several benefits, excessive collagen consumption can potentially induce the onset of diseases. Consumption of collagen peptides at high doses might lead to alterations in the gut microbiota community, while also potentially resulting in increased body weight and liver dysfunction [112]. Therefore, it is advisable to adhere to moderate collagen consumption.

Furthermore, an important collagen medical application is tissue engineering. Collagen-based materials have gained recognition as a favorable option for creating an optimal mimetic bioink aimed at regenerating multiple tissues [113]. Cardiovascular diseases contribute to 31% of annual deaths, and collagen-based vascular tissue engineering substitutes have demonstrated significant potential as physiologically relevant models for studying cardiovascular therapeutic drugs and diseases [114]. The utilization of collagen-based biomaterials for various tissue engineering purposes has been explored, including bone, cartilage, skin, dental, cornea, and urological applications [115]. While collagen-based biomaterials may not offer the mechanical support typically required for bone regeneration scenarios, the excellent biological properties of collagen type I and its versatility make it a valuable component in bone tissue engineering strategies [116]. Marine collagen has been increasingly recognized within the field of tissue engineering due to its numerous advantages, including exceptional biocompatibility, reduced zoonotic risks, decreased immunological concerns for patients with mammalian allergies, and fewer religious restrictions [117]. One of the initial instances of acellular scaffolds sourced from marine origins involved the creation of an artificial cornea derived from decellularized and decalcified tilapia scales [118]. Mineralized collagen scaffolds (MCSs) have gained prominence as bone substitutes in tissue engineering applications. Traditional techniques for creating MCSs involve direct mineral addition and in situ mineralization. An emerging fabrication method for MCSs involves the utilization of 3D printing [119]. Significant progress has been made in collagen for bone tissue engineering, with advances in sourcing, extraction, and modification techniques. Collagen-based composites show promise for bone regeneration but face clinical translation challenges [120]. Researchers keep exploring patient-specific materials for future applications.

## 6. Conclusions

Collagen constitutes the primary protein found within our bodies, and it is susceptible to decline with aging. The deficiency or loss of collagen might be a factor attributed to complex diseases or comorbid conditions. This study discusses multiple comorbid conditions that are linked to collagen. This study explores a variety of comorbid conditions interconnected with collagen. These diseases included RA, osteoporosis, osteoarthritis, psoriatic arthritis, sarcopenia, GERD, skin aging, diabetes mellitus, and periodontitis. Patients with these comorbid conditions could potentially benefit from collagen therapy. The collagen therapeutic approaches discussed in this paper may still be in the experimental stage, or some of them may have already been applied in the clinic. The current status of these therapeutic approaches needs more updated information. Moderate consumption of nutrients or supplements for collagen synthesis is recommended for aging individuals and patients with collagen deficiency. 

## Figures and Tables

**Table 1:** The tissues or organs composed of the five predominant types of collagen.

Collagen	α-Chains	Tissue or Organ
Type I	${\left[\alpha 1\left(I\right)\right]}_{2}\alpha 2$(I)	skin, bone, teeth, tendons, ligaments, vascular ligature
Type II	${\left[\alpha 1\left(II\right)\right]}_{3}$	cartilage
Type III	${\left[\alpha 1\left(III\right)\right]}_{3}$	muscle, blood vessels
Type IV	${\left[\alpha 1\left(IV\right)\right]}_{2}\alpha 2$(IV) ${\left[\alpha 3\left(IV\right)\right]}_{2}\alpha 4$(IV) ${\left[\alpha 5\left(IV\right)\right]}_{2}\alpha 6$(IV)	basal lamina, the epithelium-secreted layer of the basement membrane
Type V	$\alpha 1\left(V\right),\alpha 2\left(V\right),\alpha 3(V)$	hair, cell surfaces, placenta, skin, tendons, ligaments

## Data Availability

Not applicable.
